# Socio-Economic Determinants of Access to Orthodontic Treatment: A Cross-Sectional Study in the Romanian Population

**DOI:** 10.3390/dj14070404

**Published:** 2026-07-03

**Authors:** Olimpia Bunta, Doina Jizdan, Gabriela Ofelia Chiciudean, Daniel Ioan Chiciudean, Dana Festila

**Affiliations:** 1 Orthodontics Department, Faculty of Dental Medicine, Iuliu Hatieganu University of Medicine and Pharmacy, 400012 Cluj-Napoca, Romania; 2 Economic Sciences, Faculty of Horticulture and Business in Rural Development, University of Agricultural Sciences and Veterinary Medicine, 400372 Cluj-Napoca, Romania; daniel.chiciudean@usamvcluj.ro

**Keywords:** orthodontics, socioeconomic factors, health services accessibility, oral health, health knowledge, attitudes, practice, healthcare disparities, cross-sectional studies, Romania

## Abstract

**Background:** Malocclusion has important functional, esthetic, and psychosocial consequences; however, access to orthodontic treatment remains uneven and strongly influenced by socio-economic factors. While these disparities are well documented internationally, evidence from Romania remains limited. This study aimed to evaluate the influence of socio-economic factors on orthodontic treatment initiation within the Romanian population. **Methods:** A cross-sectional questionnaire-based study was conducted in 2025 using an online survey distributed through social media and community networks. A total of 285 adults were included. Data were analyzed using descriptive statistics, chi-square tests, and multivariable logistic regression. **Results:** Overall, 56.5% of respondents reported having undergone orthodontic treatment. Age and self-perceived information level were significantly associated with treatment initiation in the multivariable model. Participants older than 30 years were significantly less likely to have undergone orthodontic treatment compared with those aged 18–30 years (OR = 0.28, 95% CI: 0.12–0.62, *p* = 0.002). Higher levels of self-perceived information were associated with a greater likelihood of having undergone orthodontic treatment (OR = 0.75, 95% CI: 0.59–0.96, *p* = 0.020). Income and area of residence were not significantly associated with treatment initiation. However, respondents with lower income levels were significantly more likely to perceive treatment cost as a barrier to orthodontic care. **Conclusions:** Within this surveyed sample, age and self-perceived information level were independently associated with orthodontic treatment initiation. Although income was not associated with treatment uptake, financial cost remained an important perceived barrier, particularly among lower-income respondents. Given the convenience sampling strategy and limited representativeness of the sample, the findings should be interpreted as exploratory and require confirmation in larger population-based studies.

## 1. Introduction

Malocclusion negatively affects oral health by impairing mastication, swallowing, speech, and oral self-care, and contributes to temporomandibular disorders, joint pain, and accelerated tooth wear. Over time, these problems may lead to periodontal disease, caries, and systemic complications related to poor nutrition or chronic pain [1]. Beyond functional consequences, malocclusion alters facial balance and negatively affects self-esteem and social integration, particularly in children and adolescents, who are highly sensitive to esthetic and psychosocial factors [2,3]. In a society where facial esthetics are closely linked to social and professional success, misaligned teeth may lead to stigma, social anxiety, and integration difficulties, especially during adolescence [3].

Access to orthodontic treatment is strongly influenced by socio-economic and environmental factors, particularly income, place of residence, and level of information. Personal income is a major determinant, as the high cost and long duration of orthodontic treatment represent significant financial barriers. Individuals with higher incomes are more likely to initiate treatment, while lower-income families often prioritize essential expenses and perceive orthodontic care as a luxury rather than a necessity. Limited public subsidies and insufficient insurance coverage further widen social disparities [1,4]. Financial constraints are consistently identified as the main barrier to treatment initiation, with 34.5% of respondents reporting cost as the primary reason for not starting treatment [5]. Recent evidence continues to demonstrate that financial affordability remains a major determinant of orthodontic treatment uptake. Chaturvedi et al. (2024) reported that treatment cost and limited financial resources remain among the most frequently cited barriers to accessing orthodontic care, emphasizing the need for policies aimed at im-proving affordability and reducing economic inequalities in access to treatment [6]. Furthermore, population-based data from Norway showed that public reimbursement schemes may reduce socio-economic disparities in orthodontic service utilization, highlighting the importance of healthcare financing structures in promoting equitable access [7]. These disparities contribute to more severe untreated dental conditions in rural areas [8].

The level of information and education significantly influences attitudes toward orthodontic treatment. Better-informed individuals are more likely to recognize the functional and psychosocial benefits of treatment and to perceive orthodontic care as a necessity. Higher educational levels are associated with increased acceptance of orthodontic care, and well-educated parents are more willing to invest time and financial resources in their children’s treatment [9]. More recently, oral health literacy has emerged as an important determinant of treatment-seeking behavior. Individuals with greater knowledge and understanding of oral health conditions are more likely to recognize orthodontic treatment needs and engage with preventive and corrective dental services [10].

Despite extensive international research confirming the impact of socio-economic factors on access to orthodontic treatment, there is a notable lack of population-based studies addressing these issues in Romania. In contrast to Nordic countries, where public funding facilitates access to orthodontic care, countries in Eastern Europe, including Romania, continue to face financial, systemic, and geographic barriers that limit treatment availability and contribute to pronounced urban–rural disparities [11]. However, the extent to which income, area of residence, and level of information interact to influence orthodontic treatment decisions within the Romanian population remains insufficiently explored.

Moreover, evidence regarding cultural influences, patient compliance, and long-term psychosocial outcomes remains inconsistent. While orthodontic treatment has been shown to improve self-esteem [2], these benefits appear less pronounced in adults [12]. Although modern esthetic appliances such as clear aligners are perceived as more acceptable, their clinical effectiveness is comparable to traditional fixed appliances at a substantially higher cost [13]. The relationship between socio-economic status and compliance also remains debated, with some studies associating disadvantaged backgrounds with lower compliance [14], while others highlight the mitigating role of motivation and oral health education [15].

Given the limited evidence regarding socio-economic determinants of orthodontic treatment access in Romania, further research is needed to better understand how demographic, economic, and informational factors influence treatment decisions within this population.

Therefore, the aim of the present study was to evaluate the influence of socio-economic factors on access to orthodontic treatment and the decision to initiate treatment in the Romanian population. In particular, the study examined the relationship between monthly income, area of residence (urban versus rural), and level of oral health information, as well as their impact on treatment initiation, perceived barriers, and access to orthodontic care.

### Working Hypotheses

(1) Income-related hypothesis

Individuals with higher monthly incomes are significantly more likely to initiate orthodontic treatment than those with lower incomes.

(2) Cost-related hypothesis

The cost of orthodontic treatment represents the main obstacle to initiating orthodontic care, particularly among individuals with lower income levels.

(3) Environmental hypothesis

Individuals from urban areas access orthodontic treatment more frequently than those from rural areas due to differences in healthcare infrastructure and availability of specialized services.

(4) Information-related hypothesis

Individuals who perceive themselves as better informed about the functional and aesthetic benefits of orthodontic therapy may be more likely to seek treatment and to perceive it as a necessary component of oral healthcare.

## 2. Materials and Methods

### 2.1. Study Design

This study was designed as an observational, descriptive, cross-sectional investigation and was conducted in November 2025. The target population consisted of individuals aged 18 years and older who were past, present or potential future beneficiaries of orthodontic treatment. The accessible population included individuals who had access to the online platforms used for questionnaire distribution, such as social media networks and online community groups. No formal sample size calculation was performed. The study used a convenience sampling approach, and all eligible respondents who completed the questionnaire during the study period were included in the analysis.

### 2.2. Inclusion Criteria

Inclusion criteria were:

- Individuals aged 18 years or older;
- Voluntary participation, indicated by implicit informed consent through questionnaire completion;
- Answered all mandatory questions.

### 2.3. Exclusion Criteria

Exclusion criteria included:

- Individuals younger than 18 years;
- Incomplete questionnaires (did not answer the mandatory questions);
- Responses suspected to have logical inconsistencies.

### 2.4. Data Collection Instrument

Data were collected using an electronic questionnaire developed on the Google Forms platform (Google LLC, Mountain View, CA, USA). The questionnaire (File S1) comprised 23 items, including 20 single- or multiple-choice questions and three questions using a 5-point Likert scale and was organized into five sections: (1) socio-demographic characteristics, (2) experience with orthodontic treatment, (3) accessibility and economic factors, (4) treatment duration and general perceptions, and (5) information level and decision-making. The questionnaire was developed by adapting items from previously published studies investigating orthodontic treatment access, socio-economic determinants of treatment utilization, and patient perceptions regarding orthodontic care. The selected items were reviewed and modified to reflect the objectives of the present study and the Romanian context. The final questionnaire consisted primarily of closed-ended questions addressing socio-demographic characteristics, orthodontic treatment experience, perceived barriers, treatment perceptions, and self-perceived information level. The items included in sections 2–5 were adapted from previously published studies and established instruments [16,17,18,19,20], which served as conceptual references during questionnaire development. However, the final adapted questionnaire was not subjected to formal psychometric validation, pilot testing, or content validity assessment prior to implementation. Because the questionnaire was designed to assess multiple independent domains rather than a single latent construct, internal consistency reliability testing was not considered appropriate for the instrument as a whole. The questionnaire was designed to assess socio-demographic variables (age, sex, income, occupation, and residential environment), history of orthodontic treatment, perceptions of treatment effectiveness and self-image, and financial and informational accessibility to orthodontic care. Although the questionnaire item was originally labeled “gender”, it assessed biological sex (male/female) rather than gender identity; therefore, the term “sex” is used throughout the manuscript.

All data were collected anonymously, and no identifying information was recorded. The study followed the ethical principles of the Declaration of Helsinki and the study protocol was approved by the Ethics Committee of the “Iuliu Hatieganu” University of Medicine and Pharmacy (approval 2317/2025). Participation was voluntary and anonymous, and implicit informed consent was obtained from all participants through acceptance of questionnaire completion.

The questionnaire was electronically disseminated using WhatsApp (WhatsApp Ireland Limited, Dublin, Ireland), Facebook and Instagram (Meta platforms, Menlo Park, CA, USA) to 323 potential respondents who met the inclusion criteria. This study was reported in accordance with the STROBE guidelines for cross-sectional studies. The completed STROBE checklist is provided in the Supplementary Materials (File S2).

### 2.5. Statistical Analysis

The collected data were exported to Microsoft Excel (Microsoft, Redmond, Washington, DC, USA) and subsequently analyzed using IBM SPSS Statistics version 27 (IBM Corp., Armonk, NY, USA) and Microsoft Excel software. Statistical analysis included calculating descriptive statistics (frequencies, percentages, and means), bivariate analyses, and multivariable logistic regression analysis. Associations between categorical variables were evaluated using contingency tables and the chi-square (χ^2^) test. The chi-square test was applied to assess whether statistically significant differences existed between variable distributions, such as differences in cost perception between urban and rural respondents. This method enabled the identification of relevant relationships between socio-economic factors (income, area of residence, and level of information) and behaviors or perceptions related to orthodontic treatment. A multivariable binary logistic regression analysis was performed to identify independent predictors of orthodontic treatment initiation. Variables included in the model were sex, age group, area of residence, occupation, income level, and self-perceived information level. Reference categories were male sex, urban residence, age 18–30 years, and no income. For self-perceived information level, the odds ratio represents the change associated with a one-point increase on the 5-point Likert scale. The dependent variable was orthodontic treatment initiation (yes/no). In the logistic regression model, the non-treatment category was specified as the outcome event. Therefore, odds ratios below 1 indicate lower odds of belonging to the non-treatment group and consequently a greater likelihood of having undergone orthodontic treatment. Statistical significance was set at *p* < 0.05, with values below this threshold indicating that the observed associations were unlikely to be due to chance.

## 3. Results

### 3.1. Socio-Demographic Characteristics of Respondents

A total of 285 participants fully completed the mandatory questions in the questionnaire and were included in the analysis. Females represented the majority of respondents (72.3%), while males accounted for 27.7% of the sample.

**Figure 1:**
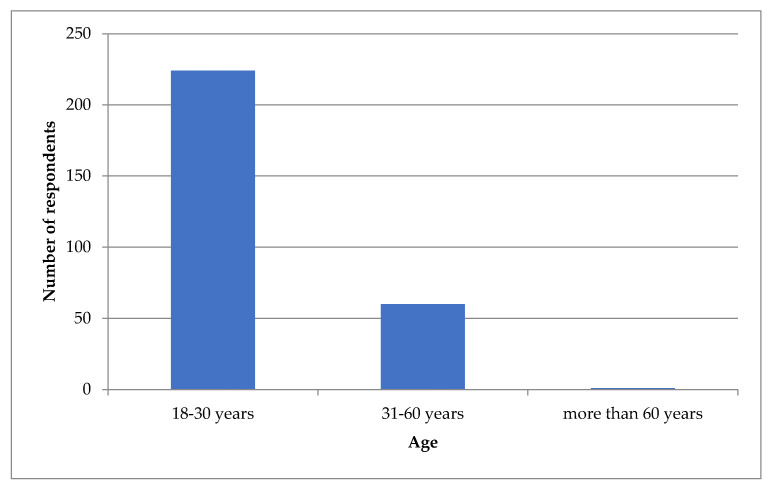
Age distribution of study participants (n = 285). Younger adults aged 18–30 years represented the majority of respondents (78.6%), followed by participants aged 31–60 years (21.1%) and those older than 60 years (0.4%).

Most participants were young adults aged 18–30 years (78.6%), followed by individuals aged 31–60 years (21.1%). Only one respondent (0.4%) was older than 60 years (Figure 1).

The majority of participants lived in urban areas (81.4%), whereas 18.6% resided in rural areas.

Regarding occupation, students represented the largest group (55.1%), followed by employees (33.7%), self-employed individuals (6.0%), entrepreneurs (4.6%), and retirees (0.7%).

Overall, 161 respondents (56.5%) reported undergoing or having undergone orthodontic treatment, while 124 participants (43.5%) had never initiated such treatment (Table 1).

### 3.2. Association Between Socio-Demographic Variables and Orthodontic Treatment Initiation

Chi-square tests were performed to evaluate the association between socio-demographic factors and orthodontic treatment initiation (Table 2).

No statistically significant association was observed between gender and orthodontic treatment initiation (χ^2^ = 1.53, *p* = 0.217).

Age was significantly associated with orthodontic treatment initiation (χ^2^ = 20.68, *p* < 0.001). Younger respondents reported a higher prevalence of orthodontic treatment compared with older participants.

Occupation was also significantly associated with treatment initiation (χ^2^ = 11.50, *p* = 0.022), with students reporting the highest proportion of orthodontic treatment experience.

No statistically significant associations were observed between residence environment and treatment initiation (χ^2^ = 0.81, *p* = 0.367) or between income level and orthodontic treatment initiation (χ^2^ = 5.28, *p* = 0.260).

### 3.3. Logistic Regression Analysis

A multivariate logistic regression analysis was conducted to evaluate the combined influence of socio-demographic and informational factors on orthodontic treatment initiation (Table 3). The model was statistically significant (χ^2^ = 30.786, *p* = 0.001) and explained approximately 14.7% of the variance in treatment initiation (Nagelkerke R^2^ = 0.147).

Age and self-perceived information level were statistically significant predictors of orthodontic treatment initiation. Participants older than 30 years were significantly less likely to have undergone orthodontic treatment compared with those aged 18–30 years (OR = 0.28, 95% CI: 0.12–0.62, *p* = 0.002). Higher levels of self-perceived information regarding orthodontic treatment were independently associated with orthodontic treatment initiation (OR = 0.75, 95% CI: 0.59–0.96, *p* = 0.020). Because the non-treatment category was modeled as the outcome event, this odds ratio indicates a lower likelihood of belonging to the non-treatment group and therefore a greater likelihood of having undergone orthodontic treatment. Sex (*p* = 0.431), residence environment (*p* = 0.942), occupation (*p* = 0.485), and income level (*p* = 0.545) were not significantly associated with orthodontic treatment initiation in the multivariate model.

**Hypothesis** **1.** *There is an association between income and orthodontic treatment initiation.*

A cross-tabulation analysis was conducted to examine the relationship between monthly income and orthodontic treatment initiation. The chi-square test did not reveal a statistically significant association between these variables (χ^2^ = 5.28, *p* = 0.260). Although respondents belonging to higher income categories appeared slightly more likely to report previous orthodontic treatment experience, the differences observed between income groups were not statistically significant. Consequently, the income-related hypothesis was not supported by the statistical analysis in the present sample.

Previous studies have reported a strong relationship between socio-economic status and orthodontic treatment utilization, suggesting that individuals with higher income levels are more likely to access orthodontic care [21,22]. However, the lack of a significant association in the present study may reflect the demographic characteristics of the sample, which included a high proportion of young adults and students who may rely on family financial support rather than personal income.

**Hypothesis** **2.** *Cost is a barrier to orthodontic treatment.*

The association between income level and the perception of treatment cost as a barrier to orthodontic treatment was evaluated using the chi-square test. The analysis revealed a highly statistically significant relationship (*p* < 0.001), indicating that respondents with lower income levels were more likely to perceive financial cost as a major obstacle to initiating orthodontic care. These findings support the hypothesis that treatment cost represents an important barrier to orthodontic treatment, particularly among individuals with limited financial resources.

Similar observations have been reported in previous studies investigating barriers to orthodontic care. Research conducted in different populations has consistently identified treatment cost as one of the primary factors influencing patients’ decisions to initiate orthodontic therapy [16,18,19]. The strong association observed in the present study further highlights the significant influence of financial constraints on treatment decision-making.

**Hypothesis** **3.** *Area of residence influences orthodontic treatment initiation.*

The relationship between area of residence (urban versus rural) and orthodontic treatment initiation was analyzed using cross-tabulation and chi-square testing. The results did not demonstrate a statistically significant association between these variables (χ^2^ = 0.81, *p* = 0.367). Although respondents living in urban environments reported slightly higher treatment initiation rates compared with those residing in rural areas, the observed differences were not statistically significant in the present sample. Therefore, the environmental hypothesis was not supported by the statistical analysis.

Nevertheless, previous studies have frequently reported disparities in orthodontic treatment access between urban and rural populations [23]. Limited availability of orthodontic specialists, greater travel distances, and reduced access to healthcare infrastructure have been identified as common barriers affecting rural communities [24]. These findings suggest that although area of residence was not significantly associated with treatment initiation in the present study, geographic factors may still influence perceptions of treatment accessibility and healthcare utilization.

**Hypothesis** **4.** *There is an association between level of information and treatment initiation.*

The relationship between self-perceived oral health knowledge and orthodontic treatment initiation was evaluated using the chi-square test. A statistically significant association was identified (*p* = 0.001), indicating that individuals who perceived themselves as better informed about orthodontic treatment options were more likely to initiate treatment. These findings are consistent with previous research demonstrating that awareness and access to reliable information represent important determinants of orthodontic treatment acceptance. Individuals who understand the functional and esthetic benefits of orthodontic therapy are more likely to seek treatment and to perceive it as a necessary component of oral healthcare [20].

Furthermore, self-perceived information level remained significantly associated with orthodontic treatment initiation after adjustment for age, sex, area of residence, occupation, and income in the multivariable logistic regression model (OR = 0.75, 95% CI: 0.59–0.96, *p* = 0.020). Detailed cross-tabulations of self-perceived information level and orthodontic treatment initiation are presented in Supplementary Table S1.

### 3.4. Additional Statistical Analysis

Several additional associations were explored to further understand perceptions and barriers related to orthodontic treatment.

Perceptions regarding the accessibility of orthodontic treatment differed significantly according to area of residence (χ^2^ = 12.99, *p* = 0.005). Respondents living in rural areas more frequently reported uncertainty regarding treatment accessibility, whereas urban participants more often considered orthodontic treatment accessible, mainly to individuals with higher income levels.

A statistically significant association was also observed between area of residence and expected orthodontic treatment duration (χ^2^ = 20.66, *p* < 0.001). Urban respondents more frequently indicated the typical treatment duration of 1–2 years, while rural respondents more frequently reported uncertainty regarding treatment duration.

Finally, perceived barriers to orthodontic treatment differed significantly across age groups (χ^2^ = 54.03, *p* < 0.001). Treatment cost was identified as the most frequently reported barrier across all age categories, particularly among respondents aged 19–30 years.

## 4. Discussion

The present study investigated the influence of socio-economic factors on access to orthodontic treatment and the decision to initiate treatment within the Romanian population. The findings highlight the role of demographic, informational, and perceived economic factors in shaping orthodontic treatment behaviors. In particular, age emerged as the strongest predictor of orthodontic treatment initiation, while perceived treatment cost represented the most commonly reported barrier to orthodontic care.

Age and self-perceived information level remained statistically significant predictors of orthodontic treatment initiation in the multivariate logistic regression model. Younger respondents were significantly more likely to report previous orthodontic treatment compared with older individuals. This finding may reflect the increasing importance of dental esthetics and facial appearance among younger generations, as well as greater exposure to orthodontic information through social media, healthcare campaigns, and routine dental care. Previous research has also demonstrated that adolescents and young adults tend to show greater interest in orthodontic treatment due to esthetic concerns and psychosocial motivations [2]. However, the long-term psychosocial benefits of orthodontic treatment may be less pronounced in adults, which could partially explain the lower treatment prevalence observed in older age groups [12].

Although previous studies have reported associations between socio-economic status and orthodontic treatment utilization, the absence of such an association in the present study may reflect the demographic characteristics of the sample, particularly the high proportion of young adults and students who may rely on parental financial support rather than personal income. Earlier research has suggested that individuals with higher socio-economic status are more likely to access orthodontic care due to greater financial resources and healthcare access [1,4]. Similarly, large population-based studies have reported associations between income inequality and orthodontic treatment utilization [21,22]. Recent evidence further supports the influence of socio-economic context on orthodontic service utilization. Jiang et al. demonstrated that although public subsidies may attenuate inequalities, socio-economic gradients in treatment access persist, particularly among disadvantaged groups. These findings suggest that structural and financial factors continue to influence access to orthodontic care even in healthcare systems with reimbursement mechanisms [7]. The absence of a statistically significant relationship in the present study may be explained by the demographic characteristics of the sample, which included a high proportion of young adults and students who may rely on parental financial support rather than personal income.

Although income was not associated with treatment initiation, respondents with lower income levels were significantly more likely to perceive treatment cost as a barrier to orthodontic care. Therefore, the observed effect relates to perceived treatment barriers rather than actual treatment initiation. These findings are consistent with numerous studies indicating that treatment cost represents one of the most important barriers to orthodontic care worldwide [25]. In many healthcare systems, including Romania, orthodontic treatment is largely financed privately and often requires long-term financial commitment. As a result, patients may perceive orthodontic therapy as a luxury rather than an essential component of oral healthcare.

This interpretation is supported by recent evidence indicating that affordability remains one of the strongest determinants of orthodontic treatment uptake. Chaturvedi et al. (2024) reported that treatment expenses continue to discourage many individuals from initiating orthodontic care, particularly among lower socio-economic groups, reinforcing the importance of financial accessibility in reducing treatment inequalities [6].

Geographic factors also play an important role in shaping perceptions related to orthodontic treatment. Area of residence was not independently associated with orthodontic treatment initiation; however, differences were observed in perceptions regarding treatment accessibility and expected treatment duration. Respondents living in rural areas were more likely to report uncertainty regarding treatment accessibility and treatment characteristics. These findings may reflect disparities in access to orthodontic specialists, healthcare infrastructure, and oral health information. Previous studies have similarly reported differences between urban and rural populations in terms of access to dental care and orthodontic services [8]. Geographic disparities may therefore influence treatment perceptions even when treatment initiation rates appear comparable. The observed association between self-perceived information level and orthodontic treatment initiation supports previous evidence suggesting that awareness and access to reliable oral health information may influence treatment-seeking behavior. Individuals who better understand the functional and esthetic benefits of orthodontic therapy may be more likely to perceive treatment as a necessary health intervention rather than a purely cosmetic procedure. Therefore, the observed association should be interpreted as reflecting participants’ perceptions of their knowledge rather than their objectively assessed level of oral health literacy. These findings support previous research demonstrating that awareness of treatment benefits and access to reliable health information significantly influence orthodontic treatment acceptance [9]. Recent studies have further highlighted the importance of oral health literacy and patient awareness in treatment decision-making. Higher levels of oral health literacy have been associated with improved recognition of orthodontic treatment needs and greater engagement with oral healthcare services [10]. Similarly, contemporary evidence suggests that willingness to undergo orthodontic treatment is strongly influenced by knowledge, perceived benefits, and awareness of available treatment options [10,20].

The findings of the present study should be interpreted in the context of the sampling methodology employed. Although the convenience sampling strategy limits the representativeness of the study population and restricts the generalizability of the results, the statistical analyses remain valid for the surveyed sample and provide exploratory evidence regarding potential associations between socio-economic factors and orthodontic treatment experiences. Consequently, the observed relationships should be interpreted as hypothesis-generating rather than as nationally representative estimates.

Overall, the findings of this study highlight the complex interaction between demographic characteristics, perceived financial barriers, and informational factors in determining access to orthodontic treatment. While financial constraints and geographic disparities may influence treatment perceptions, the decision to initiate orthodontic therapy appears to be influenced by multiple interacting determinants. Although exploratory in nature, these findings suggest that future public health strategies should consider the potential influence of informational, economic, and geographic factors on access to orthodontic care.

These results emphasize the importance of integrated public health strategies aimed at improving oral health education, increasing awareness of orthodontic treatment benefits, and expanding access to orthodontic services.

The relatively modest explanatory power of the regression model (Nagelkerke R^2^ = 0.147) suggests that additional psychosocial, behavioral, and clinical factors may contribute to orthodontic treatment decisions.

### 4.1. Clinical and Research Implications

The findings of this study have several potential clinical and research implications. From a clinical perspective, orthodontists and dental practitioners should be aware that perceived financial barriers and access to reliable information may influence patients’ attitudes toward orthodontic treatment. Enhanced patient education, clear communication regarding treatment benefits, and increased awareness of available treatment options may help improve treatment acceptance and informed decision-making. In addition, efforts to improve access to orthodontic services in underserved areas may help reduce perceived geographic disparities in care.

From a research perspective, future studies should employ larger and more representative samples, preferably using probability-based sampling methods, to confirm the associations observed in the present study. Further research incorporating objective measures of oral health literacy, clinical assessments of orthodontic treatment need, and psychosocial determinants of treatment-seeking behavior would provide a more comprehensive understanding of factors influencing access to orthodontic care.

### 4.2. Study Limitations

Several limitations should be considered when interpreting the findings of this study. First, the use of a self-administered online questionnaire did not allow for direct clarification of questions and may have introduced response or interpretation bias. Second, the questionnaire was distributed through social media and messaging platforms using a convenience sampling strategy. Consequently, individuals who actively use internet-based communication platforms were more likely to participate, introducing potential selection bias. Older adults, individuals with limited internet access, and socio-economically disadvantaged groups may have been underrepresented. This likely contributed to the overrepresentation of younger adults, females, students, and urban residents in the study sample. As a result, the findings cannot be considered nationally representative and should not be generalized to the Romanian population as a whole.

Third, the relatively small sample size (n = 285) may have limited the statistical power, particularly in subgroup analyses, and further restricts the representativeness of the study population. Fourth, self-reported oral health knowledge was used as a subjective measure of perceived information level and does not reflect objectively assessed oral health literacy. Therefore, findings related to Hypothesis 4 should be interpreted as reflecting participants’ self-perceived knowledge and awareness rather than objectively assessed oral health literacy, and should be interpreted with caution. Additionally, although the questionnaire was developed using items adapted from previously published instruments, the final adapted version was not subjected to formal psychometric validation, pilot testing, or content validity assessment. Therefore, measurement error and variability in item interpretation cannot be completely excluded.

Another limitation is that reporting income was optional because of the potentially intrusive nature of this question and concerns regarding participant privacy. As a result, only 264 of the 285 respondents provided valid income data, introducing the possibility of non-response bias in analyses involving economic variables.

Finally, no clinical assessment of orthodontic treatment need or malocclusion severity was performed. Consequently, the analysis focused on perceived access to orthodontic care and self-reported treatment experience rather than objectively measured orthodontic need. Future studies combining epidemiological surveys with clinical examinations would provide a more comprehensive assessment of inequalities in orthodontic treatment access.

The relatively modest explanatory power of the regression model (Nagelkerke R^2^ = 0.147) suggests that additional psychosocial, behavioral, and clinical factors not captured in the present study may contribute to orthodontic treatment decisions.

Despite these limitations, the findings contribute to the limited evidence regarding socio-economic factors associated with orthodontic treatment access in Romania. However, the results should be regarded as exploratory and hypothesis-generating and require confirmation in larger studies using more representative sampling methods.

## 5. Conclusions

Within this surveyed sample, demographic factors, particularly age, were associated with orthodontic treatment initiation, while perceived informational factors may also contribute to treatment uptake. Given the convenience sampling strategy and limited representativeness of the sample, these findings should be considered exploratory and require confirmation in larger population-based studies. Younger individuals were significantly more likely to have undergone orthodontic treatment compared with older respondents. Although income was not associated with treatment initiation, respondents with lower income levels were significantly more likely to perceive treatment cost as a barrier to orthodontic care. In addition, differences between urban and rural populations were observed regarding perceptions of treatment accessibility and treatment characteristics, although place of residence was not significantly associated with orthodontic treatment initiation. Individuals reporting higher self-perceived levels of information were more likely to initiate treatment, underscoring the potential role of oral health education. These findings suggest that improving access to orthodontic care requires a multifactorial approach addressing both economic and informational inequalities. Although exploratory in nature, the findings suggest that enhancing oral health education, addressing perceived financial barriers, and improving access to orthodontic services may contribute to reducing inequalities in access to care. Further research integrating epidemiological surveys with clinical assessments of orthodontic treatment need is necessary to provide a more comprehensive understanding of treatment disparities and to support the development of evidence-based public health policies.

## Figures and Tables

**Table 1:** Socio-demographic characteristics of respondents (n = 285).

Characteristic	Category	n	%
Gender	Female	206	72.3
	Male	79	27.7
Age group	18–30 years	224	78.6
	31–60 years	60	21.1
	≥60 years	1	0.4
Residence	Urban	232	81.4
	Rural	53	18.6
Occupation	Student	157	55.1
	Employee	96	33.7
	Self-employed	17	6.0
	Entrepreneur	13	4.6
	Retired	2	0.7
Orthodontic treatment history	Yes	161	56.5
	No	124	43.5

**Table 2:** Chi-square analysis of associations between socio-demographic characteristics, self-perceived information level, and orthodontic treatment initiation.

Variable	Category	Treatment, n (%)	No Treatment, n (%)	χ^2^	*p*-Value
Gender	Female	121 (58.7)	85 (41.3)	1.53	0.217
	Male	40 (50.6)	39 (49.4)		
Age group	18–30 years	142 (63.4)	82 (36.6)	20.68	<0.001
	31–60 years	19 (31.7)	41 (68.3)		
	≥60 years	0 (0.0)	1 (100.0)		
Residence	Urban	134 (57.8)	98 (42.2)	0.81	0.367
	Rural	27 (50.9)	26 (49.1)		
Occupation	Student	100 (63.7)	57 (36.3)	11.50	0.022
	Employee	50 (52.1)	46 (47.9)		
	Self-employed	6 (35.3)	11 (64.7)		
	Entrepreneur	5 (38.5)	8 (61.5)		
	Retired	0 (0.0)	2 (100.0)		
Income *	No incomes	61 (62.2)	37 (37.8)	5.28	0.260
	Below 400€	13 (50.0)	13 (50.0)		
	400–800€	38 (60.3)	25 (39.7)		
	801–1500€	22 (48.9)	23 (51.1)		
	More than 1500€	14 (43.8)	18 (56.3)		

Percentages are calculated within rows. * Income variable includes 264 valid responses (missing = 21).

**Table 3:** Multivariable logistic regression analysis of orthodontic treatment initiation (dependent variable) according to sex, age, area of residence, occupation, income, and self-perceived information level (independent variables).

Variable	Comparison (Reference Category)	OR (95% CI)	*p*-Value
Sex	Female vs. Male	0.78 (0.42–1.45)	0.431
Residence	Rural vs. Urban	1.03 (0.51–2.09)	0.942
Income	Overall	—	0.545
	Below 400€ vs. No income	1.11 (0.35–3.52)	0.862
	400–800€ vs. No income	1.36 (0.36–5.10)	0.650
	801–1500€ vs. No income	0.64 (0.25–1.66)	0.356
	>1500€ vs. No income	1.12 (0.42–3.00)	0.829
Self-perceived information level	Per one-point increase	0.75 (0.59–0.96)	0.020
Age	Older than 30 years (reference: 18–30 years)	0.28 (0.12–0.62)	0.002

χ^2^ = 30.786, *p* = 0.001, Nagelkerke R^2^ = 0.147. Dependent variable: orthodontic treatment initiation (1 = yes, 2 = no). The non-treatment category was specified as the outcome event; therefore, odds ratios below 1 indicate lower odds of belonging to the non-treatment group and a greater likelihood of having undergone orthodontic treatment.

## Data Availability

The raw data supporting the conclusions of this article will be made available by the authors on request.

## Supplementary Materials

The following supporting information can be downloaded at: https://www.mdpi.com/article/10.3390/dj14070404/s1, File S1. The questionnaire developed for this survey. File S2. STROBE checklist for cross-sectional studies. Table S1. Association between self-perceived information level and orthodontic treatment initiation.
