# Supplementary material for: Socio-Economic Determinants of Access to Orthodontic Treatment: A Cross-Sectional Study in the Romanian Population
Source: Dent J (Basel). 2026 Jul 3;14(7):404. doi: 10.3390/dj14070404 (PMC13408210; doi:10.3390/dj14070404)
Supplement: Supplementary file 1 [file dentistry-14-00404-s001.zip › File S1. The questionnaire developed for this survey.pdf]

QUESTIONAIRE

* Indicates a mandatory question

I. Personal data

1. What is your gender? *
   ☐Female
   ☐Male
2. What is your age? *
   ☐18-30 years
   ☐31-60 years
   ☐over 60 years old
3. What is your residence background? *
   ☐Urban
   ☐Rural
4. What do you do for a living? *
   ☐Pupil/student
   ☐Employee
   ☐Self-employed
   ☐Entrepreneur
   ☐Retired
5. What is your monthly income? (optional)
   ☐no incomes
   ☐under 2000 RON (under 400 EUR)
   ☐2000-4000 RON (400-800 EUR)
   ☐4000-6000 RON (800-1200 EUR)
   ☐over 6000 RON (over 1200 EUR)

II. Experience with orthodontic treatments

6. Have you ever undergone an orthodontic treatment? *
   ☐Yes
   ☐No
7. What is the main reason for having chosen/ choosing to undergo an orthodontic treatment? *
   ☐Esthetical consideration
   ☐Bite problems
   ☐Medical recommendation
   ☐Other reasons
   ☐I would not undergo an orthodontic treatment
8. I you ever undergone an orthodontic treatment, what were the main benefits?
   ☐Straightening teeth
   ☐Improving smile esthetics
   ☐Improving oral hygiene
   ☐Improving mastication
   ☐Improving articular pain (TMJ)
   ☐Other
9. Do you consider that an orthodontic treatment can influence self-image? *
   ☐Yes, in a positive way
   ☐Yes, in a negative way
   ☐No

☐I don’t know

10. On a scale of 1 to 5, how much do you think orthodontic treatments influence oral health? * (1- not at all; 5- very much) _______
11. If you ever underwent an orthodontic treatment, what were the major difficulties that you have faced/encountered?
    ☐Discomfort /pian after appliance application/appliance activation
    ☐Prolonged treatment time
    ☐Increased costs of treatment
    ☐Lack of information or deficient communication with the orthodontist
    ☐Masticatory problems
    ☐Difficulties in proper oral hygiene
    ☐Other

III. Accessibility and economic factors

12. On a scale of 1 to 5, how much does the cost influence your decision to undergo an orthodontic treatment? * (1- not at all; 5- very much) _______
13. If you ever underwent an orthodontic treatment, have you found the cost accessible? *
    ☐Yes
    ☐No
    ☐I did not undergo an orthodontic treatment
14. Do you think orthodontic treatments are accessible to everyone or more likely just to those with high incomes? *
    ☐They are accessible to everyone
    ☐they are more accessible to those with higher incomes
    ☐They are inaccessible to the majority of the population
    ☐I don’t know /I do not have an opinion about this aspect
15. What do you think is the main obstacle in access to orthodontic care? *
    ☐Cost
    ☐Lack of information
    ☐Distance from orthodontic providers/dental offices/dental clinics
    ☐Treatment duration
    ☐Other reasons
16. Has it ever occurred to you to give up on the first recommendation to undergo orthodontic treatment from financial reasons? *
    ☐Yes, I gave up due to high costs
    ☐No, I have undergone the initially recommended orthodontic treatment
    ☐No, I chose a more financially accessible option
    ☐No, I did not have any cost related problems
    ☐It was not the case (I did not receive a previous recommendation)
17. What are the factors that would convince you to undergo orthodontic treatment, even if the costs would be higher? (multiple answers) *
    ☐Guaranteed visible results
    ☐Doctor recommendation
    ☐Possibility of payment in instalments
    ☐Clear information regarding the benefits
    ☐Other

IV. Treatment duration and general perceptions

18. What is the orthodontic treatment duration you would expect, regardless of whether you have undergone orthodontic treatment? *

☐Under 6 months
☐6 months-1 year
☐1-2 years
☐More than 3 years
☐I don't know/ I did not think about this aspect

19. What do you think about the idea of undergoing an orthodontic treatment that lasts 2-3 years?

☐It is a normal and justified treatment duration for obtaining a good result
☐It is a long treatment duration, but acceptable if the results are visible
☐It is too long and would discourage most patients
☐I would not accept such a long treatment duration
☐I don't know/ I do not have an opinion about this aspect

20.What is your general perception about orthodontic treatments?

☐They are very necessary
☐they are necessary
☐They are not important
☐I don't know

V. Information and decision

21. On a scale of 1 to 5, how informed you consider being related to your options related to orthodontic treatment? * (1- not informed at all; 5- very informed) _______

22. Where did you/would you obtain information about orthodontic treatments? (multiple answers) *

☐Dentist
☐Internet
☐Family/friends
☐Advertisements
☐Dental clinics
☐Other

23. What were your main reasons for postponing or not starting orthodontic treatment? *

☐Costs
☐Treatment duration
☐Fear
☐Lack of information
☐Other
☐I did not postpone orthodontic treatment
