# Supplementary material for: Socio-Economic Determinants of Access to Orthodontic Treatment: A Cross-Sectional Study in the Romanian Population
Source: Dent J (Basel). 2026 Jul 3;14(7):404. doi: 10.3390/dj14070404 (PMC13408210; doi:10.3390/dj14070404)
Supplement: Supplementary file 1 [file dentistry-14-00404-s001.zip › Table S1. Association between self-perceived information level and orthodontic treatment initiation.pdf]

Supplementary

Table S1. Association between self-perceived information level and orthodontic treatment initiation.

Self-perceived information level	No orthodontic treatment n (%)	Previous orthodontic treatment n (%)	Total
Very uninformed	7 (46.7)	8 (53.3)	15
Uninformed	18 (43.9)	23 (56.1)	41
Neutral	48 (49.5)	49 (50.5)	97
Informed	44 (69.8)	19 (30.2)	63
Very informed	44 (63.8)	25 (36.2)	69
Total	161 (56.5)	124 (43.5)	285

Chi-square analysis: Pearson $\chi^2 = 11.225$, df = 4, $p = 0.024$.
Linear-by-linear association: $p = 0.006$.

Values are presented as number (row percentage). Percentages represent the distribution of orthodontic treatment history within each self-perceived information level category. Statistical significance was assessed using Pearson's chi-square test.
